# Acid Sphingomyelinase Activity in Dried Blood Spot from Neonatal Intensive Care Unit–Admitted Neonates: A Pilot Study for Expanded Newborn Screening in Japan

**DOI:** 10.3390/ijns12020022

**Published:** 2026-04-01

**Authors:** Akie Kato, Atsuko Noguchi, Hiroyuki Adachi, Kiichi Takahashi, Masato Ito, Tomoo Ito, Shozo Ota, Hirokazu Arai

**Affiliations:** 1 Department of Pediatrics, Akita University Graduate School of Medicine, Akita 010-8543, Japan; 2 Department of Neonatology, Japanese Red Cross Akita Hospital, Akita 010-1495, Japan

**Keywords:** acid sphingomyelinase deficiency, newborn screening, dried blood spot, NICU, hematocrit, developmental factors, tandem mass spectrometry, Niemann–Pick disease

## Abstract

Acid sphingomyelinase deficiency (ASMD) is currently treatable with olipudase alfa, increasing the need for early newborn screening (NBS). We conducted a two-center pilot cohort study to characterize dried blood spot (DBS) acid sphingomyelinase (ASM) activity in Japanese neonates in the neonatal intensive care unit (NICU). ASM activity was measured by flow injection-tandem mass spectrometry in 244 NICU-admitted neonates (gestational age 25–41 weeks; birth weight 773–4201 g); longitudinal paired samples were available in 34 neonates with birth weight < 2000 g and concurrent hematology in 43 neonates. The mean ASM activity was 3.7 ± 1.2 μmol/h/L (95% confidence interval, 3.54–3.84; range, 1.7–11.6), with a right-skewed distribution. ASM activity correlated positively with birth weight (r = 0.184, *p* = 0.0039), gestational age (r = 0.219, *p* = 0.0006), and lymphocyte count (ρ = 0.394, *p* = 0.0089) and negatively with hematocrit (ρ = −0.372, *p* = 0.014). In neonates with a birth weight < 2000 g, ASM increased significantly on repeat sampling (mean difference, 1.60 μmol/h/L; *p* < 0.0001; Cohen’s d = 0.912). These findings support NICU-specific reference ranges, hematology-informed interpretations, repeat testing after maturation, and the use of second-tier biomarkers for ASMD NBS implementation in Japan.

## 1. Introduction

Acid sphingomyelinase deficiency (ASMD), historically classified as Niemann–Pick disease types A and B, is a lysosomal storage disorder resulting from biallelic pathogenic variants of *SMPD1* [1,2,3,4,5,6]. The loss of acid sphingomyelinase activity results in the accumulation of sphingomyelin and secondary lipids within lysosomes, producing a wide clinical spectrum, from rapidly progressive neurodegenerative type A to chronic visceral type B [1,2,3,4,5,6]. ASMD is historically considered ultra-rare (estimated incidence of 1:100,000 to 1:1,000,000 births); a recent population-based genomic analysis has suggested a markedly higher prevalence in Japan. Sako et al. estimated a carrier frequency of 1/180 and predicted an incidence of 1/128,191 in the Japanese population, indicating substantial disease underdiagnosis, particularly in individuals with milder phenotypes [7].

The therapeutic landscape has transformed dramatically with the approval of recombinant human acid sphingomyelinase (Xenpozyme) in 2022. Enzyme replacement therapy has demonstrated sustained clinical improvements in hepatosplenomegaly, pulmonary function, hematological parameters, lipid profiles, and quality of life in both children and adults [8,9,10,11,12,13,14,15]. Early treatment initiation is associated with superior long-term outcomes, creating an urgent need for early diagnosis using newborn screening (NBS).

Dried blood spot (DBS)-based acid sphingomyelinase (ASM) activity assays offer practical advantages for NBS, such as low cost, scalability, and use of existing screening infrastructure. Pilot programs in the United States, Italy, and other regions have demonstrated their feasibility and clinical utility [16,17,18,19,20,21,22]. However, DBS-based ASM measurements are influenced by multiple factors, such as hematocrit, leukocyte composition, storage conditions, and transfusion history [3,23,24,25,26,27]. These factors are particularly important in the neonatal intensive care unit (NICU) environment, where neonates commonly experience premature birth, hematological instability, acute systemic illness, rapid physiological changes, and complex medical interventions.

However, no NICU-specific reference ranges or interpretation algorithms exist for DBS-based ASM activity. This knowledge gap has direct implications for clinical practice: distinguishing pseudodeficiency from true enzyme deficiency; establishing accurate, population-specific cutoff values; minimizing false-positive and false-negative results; and determining appropriate follow-up strategies for NICU neonates.

We hypothesized that developmental factors (gestational age and birth weight) and hematological parameters (white blood cell count, lymphocyte count, and hematocrit) would significantly influence DBS-based ASM activity in NICU neonates. To test this hypothesis, we conducted a pilot study characterizing ASM activity in 244 NICU-admitted neonates at two Japanese tertiary centers, quantified the relationship between ASM activity and clinical/hematological variables, assessed longitudinal changes in premature neonates, and compared assay performance using DBS in three patients who were previously diagnosed with ASMD. These data will provide foundational evidence for implementing ASMD expanded NBS in Japan, while accounting for the physiologic complexities unique to the NICU population.

## 2. Materials and Methods

### 2.1. Study Design and Ethical Approval

This observational cohort study combined cross-sectional baseline analyses with longitudinal follow-ups in a subset of neonates. The Akita University Graduate School of Medicine Ethics Committee (protocol no. 2485) and the Japanese Red Cross Akita Hospital Ethics Committee (protocol no. 2-6) approved the protocol. All procedures adhered to the Declaration of Helsinki and institutional regulations. Written informed consent was obtained from all the parents or legal guardians.

### 2.2. Study Population

Neonates admitted to the NICUs of Akita University Hospital and the Japanese Red Cross Akita Hospital between June 2020 and March 2023 were enrolled in the study. DBS for the initial ASM measurement was performed on postnatal days 4–6 in accordance with Japan’s national NBS protocol. For neonates with birth weight <2000 g, repeat DBS sampling was also performed according to the Japanese NBS protocol, at the earliest of approximately 1 month of age, attainment of body weight ≥2500 g, or NICU discharge. Three previously diagnosed patients with ASMD (type A, n = 1; type B, n = 2) served as positive controls. Their demographic and clinical characteristics are summarized in Table 1.

### 2.3. Sample Collection, Storage, and Hematological Assessment

No additional blood samples were collected for research purposes. Residual whole blood from routine clinical testing was spotted onto Whatman 903 filter paper, dried at room temperature for ≥5 h, placed in resealable plastic bags with desiccant, and stored at −80 °C until analysis. For a subset of neonates (n = 43) who underwent complete blood count testing for clinical indications at the time of DBS collection, hematological parameters (white blood cell count, absolute lymphocyte count, and hematocrit) were recorded for correlation analyses.

Although hematocrit is often available from blood gas analysis in NICU practice, blood gas–derived hematocrit and complete blood count (CBC) hematocrit are not always interchangeable. Therefore, to maximize analytical consistency, hematocrit correlations were restricted to neonates with same-day CBC-based measurements.

### 2.4. Measurement of ASM Activity

ASM activity was measured using the NeoLSD™ kit (Revvity, Turku, Finland) with flow-injection tandem mass spectrometry (FI-MS/MS) as previously reported [16,17,27]. Briefly, 3.2-mm DBS punches were incubated with an internal standard and substrate, reaction products were analyzed using a TSQ Vantage triple quadrupole mass spectrometer (Thermo Fisher Scientific, Waltham, MA, USA), and data were acquired and processed using the Xcalibur 4.2 software. The MS/MS analytical conditions are listed in Supplementary Tables S1 and S2. Because the assay was performed by FI-MS/MS without chromatographic separation, no compound-specific retention times were generated; all monitored ions were acquired within the 1-min injection window. The source settings listed in Supplementary Table S1, including the vaporizer temperature, reflect the original instrument method used for this assay. ASM activity was expressed as μmol/h/L (whole-blood equivalent in DBS extracts). All the samples were assayed in identical batches.

Quality control DBS materials (low, medium, and high activity) from the NeoLSD™ kit were used for intra-assay validation, demonstrating coefficients of variation of 3.9% (low), 6.6% (medium), and 9.7% (high), confirming assay reproducibility.

### 2.5. Clinical Data Collection

Demographic and clinical variables extracted from the medical records included gestational age, birth weight, sex, hematologic parameters, and major comorbidities (respiratory distress, congenital heart disease, infection, neonatal asphyxia, metabolic abnormalities, and chromosomal disorders). Supplementary Data S1–S3 are available on the journal website and include individual-level data for all 244 neonates (Supplementary Data S1), paired measurements for 34 neonates with birth weights <2000 g (Supplementary Data S2), and hematological parameters for 43 neonates with concurrent testing (Supplementary Data S3).

### 2.6. Statistical Analysis

#### 2.6.1. Descriptive Statistics and Distributional Assessment

ASM activity was characterized using mean, standard deviation (SD), median, and range. For the overall cohort (n = 244), 95% confidence intervals (CIs) for the mean were calculated using the t-distribution as: 95% CI = mean ± t_(0.975, n−1)_ × standard error. Normality of continuous variables was assessed using the Shapiro–Wilk test, with *p* < 0.05 indicating evidence against normality.

ASM activity was additionally compared between preterm neonates (<37 weeks of gestation) and term neonates (≥37 weeks of gestation) using Welch’s two-sample *t*-test.

#### 2.6.2. Correlation Analyses

Regarding birth weight and gestational age (n = 244), given the normal distribution of these developmental variables, Pearson’s correlation coefficients were calculated. For each correlation, 95% CIs were computed using Fisher’s z-transformation: z = arctanh(r), with standard error SE = 1/√(n − 3); 95% CI for r derived from inverse transformation of z-transformed CI boundaries.

For hematological parameters (n = 43), normality testing revealed that the white blood cell count and hematocrit were non-normally distributed (Shapiro–Wilk *p* < 0.05), whereas the lymphocyte count was normally distributed. Consequently, both Pearson and Spearman correlations were calculated for white blood cell counts and hematocrit. Spearman correlation (ρ) was used for primary interpretation when variables were non-normal. An identical Fisher z-transformation methodology was applied to calculate 95% CIs for Spearman correlations.

#### 2.6.3. Longitudinal Analysis

Paired measurements from 34 neonates with birth weights <2000 g were analyzed using: (1) Shapiro–Wilk normality testing of differences between paired measurements; (2) paired *t*-test when differences were normally distributed; (3) Cohen’s d effect size calculated as (mean difference)/(SD of difference); and (4) individual change patterns (percentage with increase, decrease, and no change).

#### 2.6.4. Significance and Software

All significance tests were two-tailed with α = 0.05. Statistical analyses were performed using GraphPad Prism version 9 and IBM SPSS Statistics for Mac version 28.

### 2.7. Screening Cutoff Definition

In this pilot study, we defined an operational screening cutoff as the 0.1st percentile of ASM activity within the NICU cohort to minimize false-positive referrals in a high-acuity population with substantial biological and pre-analytical variability. As an ASMD confirmatory testing laboratory designated by the Japanese Society for Inherited Metabolic Diseases, our center routinely uses 1.5 μmol/h/L as an institutional operational cutoff for DBS ASM activity; the percentile-based cutoff provided a conservative, cohort-derived threshold while maintaining continuity with this practice.

## 3. Results

### 3.1. Patient Characteristics

**Figure 1:**
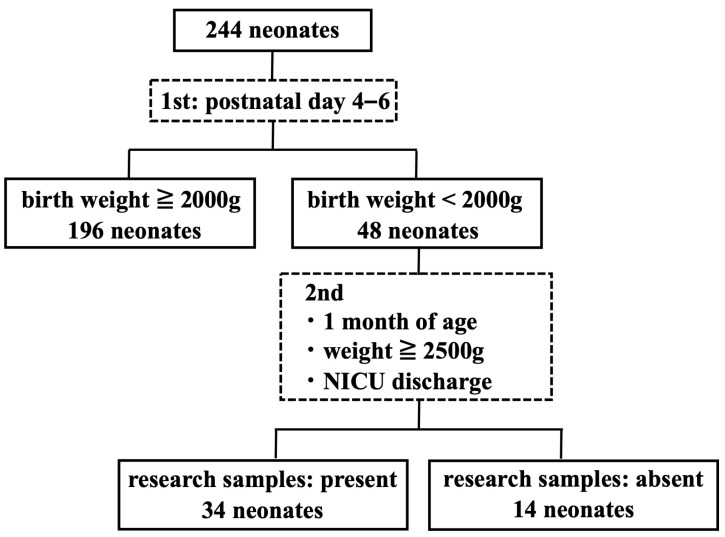
Enrollment flow and sample collection scheme for NICU-admitted neonates. Enrollment and dried blood spot (DBS) sampling flow for 244 neonates: 196 with birth weight ≥ 2000 g; 48 with birth weight < 2000 g (of whom 34 had paired DBS samples for longitudinal analysis). Initial DBS collected on postnatal days 4–6; follow-up DBS obtained at the earliest of approximately 1 month of age, weight ≥ 2500 g, or hospital discharge.

A total of 244 NICU-admitted neonates were enrolled (gestational age range, 25–41 weeks; birth weight 773–4201 g). The cohort comprised 134 male (55.0%) and 110 female (45.0%) neonates. Forty-eight neonates (19.7%) had birth weights of <2000 g, of which 34 (70.8%) had paired DBS samples for longitudinal assessment (Figure 1). Of the neonates, 146 (59.8%) were born preterm (<37 weeks of gestation). The common clinical conditions included respiratory distress, hypoglycemia, congenital heart disease, infections, hyperbilirubinemia, and chromosomal abnormalities. No neonates received a transfusion before initial DBS sampling. The clinical characteristics of the neonates are shown in Table 2.

### 3.2. Distribution of ASM Activity

#### Overall Cohort (n = 244)

**Figure 2:**
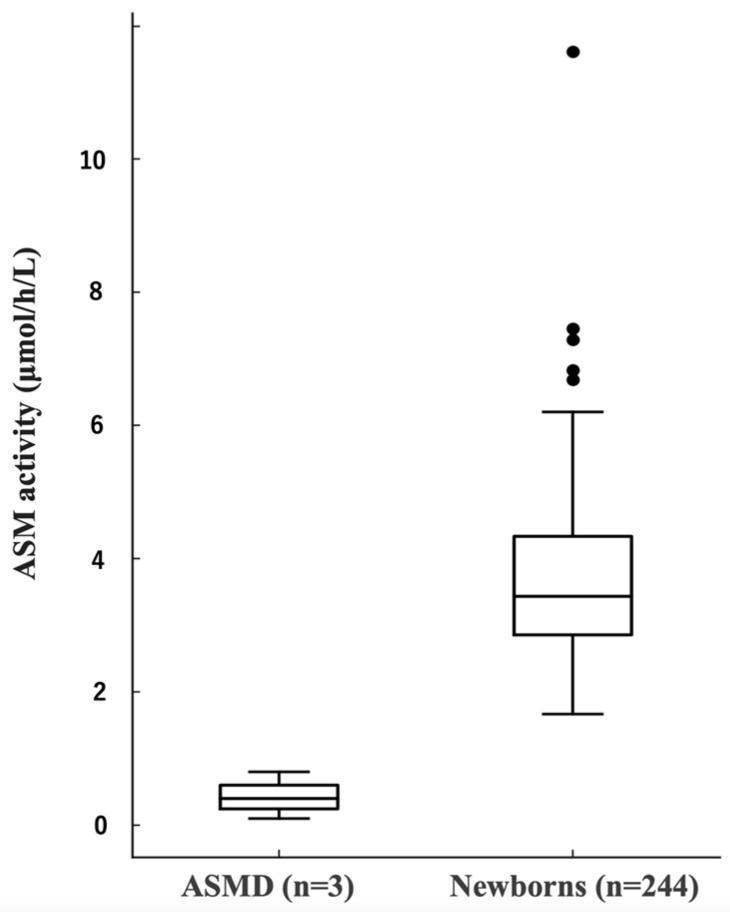
Comparison of ASM activity between patients with ASMD and NICU-admitted neonates.

The mean ASM activity was 3.7 ± 1.2 μmol/h/L (95% CI: 3.54–3.84; median, 3.4 μmol/h/L; range, 1.7–11.6 μmol/h/L). The Shapiro–Wilk test (W = 0.8899, *p* < 0.0001) indicated a non-normal distribution with a right skew. The 0.1st percentile (screening cutoff) corresponded to a 1.7 μmol/h/L. One neonate with initial value of 1.67 μmol/h/L had normalized repeat value of 2.1 μmol/h/L, confirming false positivity due to sample quality variation. The comparative distribution of ASM activity for patients with ASMD (n = 3) and neonates (n = 244) is shown in Figure 2.

Stratified analysis revealed that preterm neonates (<37 weeks, n = 146) had lower mean ASM activity (3.38 ± 0.90 μmol/h/L; 95% CI: 3.23–3.52) than term neonates (≥37 weeks, n = 98) (4.16 ± 1.42 μmol/h/L; 95% CI: 3.88–4.44). The mean difference was 0.79 µmol/h/L (95% CI: 0.47–1.11), and the between-group difference was statistically significant (Welch’s *t*-test, *p* < 0.0001). Birth weight < 2000 g (n = 48) showed similar mean ASM activity (3.62 ± 1.11 μmol/h/L; 95% CI: 3.31–3.94) to ≥2000 g (n = 196) (3.71 ± 1.23 μmol/h/L; 95% CI: 3.54–3.88).

Box-and-whisker plots: ASM activity in three patients with ASMD versus 244 NICU neonates (μmol/h/L). Screening cutoff (0.1st percentile) = 1.7 μmol/h/L. Black dots indicate outlier values beyond the whiskers. Patients with ASMD show markedly reduced enzyme activity, demonstrating a strong discriminatory performance of the assay. ASM, acid sphingomyelinase; ASMD, acid sphingomyelinase deficiency.

### 3.3. Correlations with Clinical and Hematological Variables

#### 3.3.1. Birth Weight and Gestational Age (n = 244)

**Figure 3:**
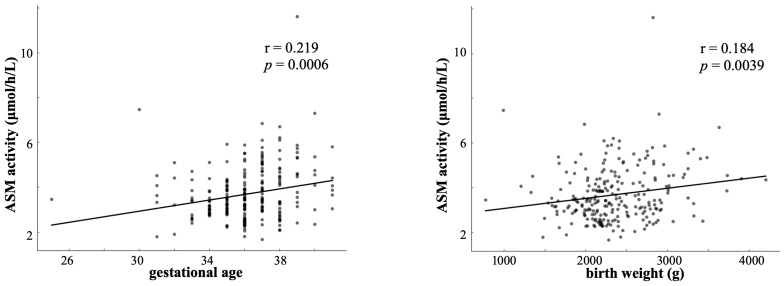
Correlation between ASM activity and developmental indicators in neonates.

ASM activity demonstrated statistically significant positive correlations with (Figure 3):

- Birth weight: Pearson r = 0.184 (95% CI: 0.060–0.303, *p* = 0.0039; n = 244). For every 1000 g increase in birth weight, ASM activity increased by approximately 0.22 μmol/h/L.
- Gestational age: Pearson r = 0.219 (95% CI: 0.096–0.335, *p* = 0.0006; n = 244). For every 1-week increase in gestational age, ASM activity increased by approximately 0.21 μmol/h/L.

These modest but consistent correlations indicate developmental influences on ASM activity, although the wide confidence intervals reflect substantial individual variations.

Scatter plots with regression lines: ASM activity vs. gestational age (left) and birth weight (right) in 244 neonates. Gestational age: Pearson’s r = 0.219, *p* =0.0006; birth weight: Pearson’s r = 0.184, *p* = 0.0039). Positive correlations indicate developmental influences on enzyme activity. ASM, acid sphingomyelinase

#### 3.3.2. Hematological Parameters (n = 43)

Normality testing showed that the ASM activity (*p* = 0.1075) and lymphocyte count (*p* = 0.9339) were normally distributed, whereas the white blood cell count (*p* = 0.0009) and hematocrit (*p* = 0.0200) were non-normally distributed.

White blood cell count showed no significant correlation with ASM activity (Pearson r = 0.085, *p* = 0.589; Spearman ρ = 0.207, *p* = 0.183).

**Figure 4:**
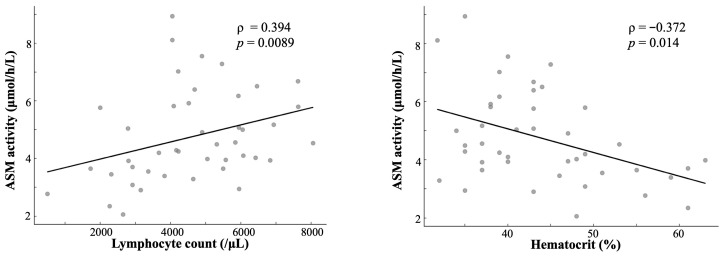
Correlation between ASM activity and hematological parameters.

Lymphocyte count showed significant positive correlation (Pearson r = 0.324, *p* = 0.034; Spearman ρ = 0.394, 95% CI: 0.107–0.621, *p* = 0.0089), suggesting that lymphocyte-associated lysosomal enzyme activity influences DBS ASM measurements (Figure 4).

Hematocrit values revealed a significant inverse correlation (Pearson r = −0.418, *p* = 0.005; Spearman ρ = −0.372, 95% CI: −0.605 to −0.081, *p* = 0.014), indicating that erythrocytes lack ASM activity. Higher hematocrit values reduced the relative white blood cell proportion per DBS punch (Figure 4).

Scatter plots of ASM activity versus lymphocyte count (left) and hematocrit (right) in 43 neonates. Lymphocyte: Spearman ρ = 0.394, *p* = 0.0089; hematocrit: Spearman ρ = −0.372, *p* = 0.014. A positive lymphocyte correlation and an inverse hematocrit correlation indicated a hematological influence on DBS enzyme measurements. ASM, acid sphingomyelinase; DBS, dried blood spot.

### 3.4. Longitudinal Analysis in Neonates with Birth Weight <2000 g (n = 34)

**Figure 5:**
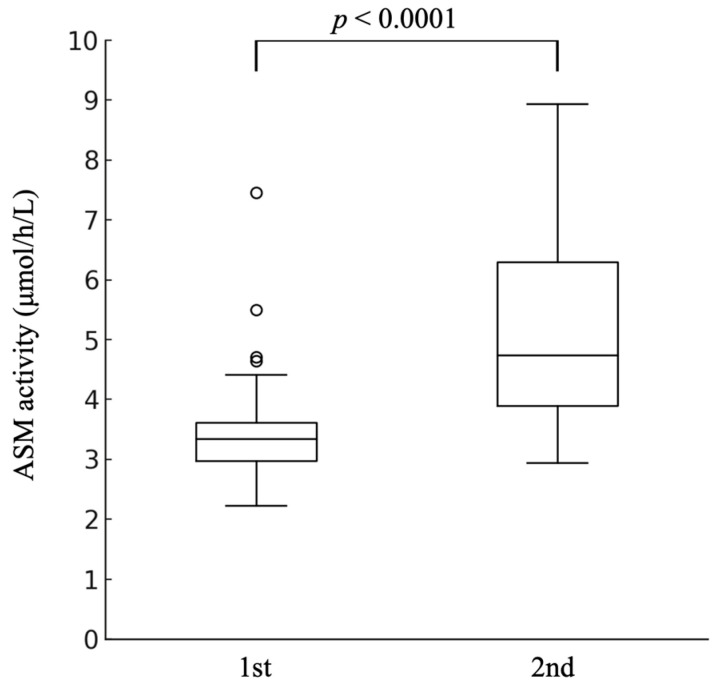
Longitudinal changes in ASM activity among neonates with birth weight < 2000 g.

Among 34 neonates with birth weights <2000 g and paired measurements, ASM activity increased significantly between the initial (postnatal days 4–6) and follow-up measurements (Figure 5).

- Initial: 3.48 ± 1.02 μmol/h/L [95% CI: 3.14–3.83];
- Follow-up: 5.09 ± 1.57 μmol/h/L [95% CI: 4.56–5.61];
- Mean difference: 1.60 ± 1.76 μmol/h/L [95% CI: 1.01–2.19].

The differences were normally distributed (Shapiro–Wilk W = 0.9604, *p* = 0.251). Paired *t*-test results: *t* = 5.32, *p* < 0.0001, Cohen’s d = 0.912 (significant effect). Individual responses: 31 neonates (91.2%) showed increased activity, and three (8.8%) showed decreased activity. The mean percentage increase was 54.2% (median, 44.3%).

Paired measurements from 34 neonates with birth weight <2000 g. ASM activity increased significantly from initial measurement (mean 3.48 μmol/h/L, postnatal days 4–6) to follow-up (mean 5.09 μmol/h/L). Open circles indicate outlier values beyond the whiskers. Paired *t*-test: *t* = 5.32, *p* < 0.0001; Cohen’s d = 0.912; 91.2% showed improvement; the mean percentage increase was 54.2%.

### 3.5. ASMD Case Identification

None of the neonates exhibited persistent ASM deficiency below the 1.7 μmol/h/L cutoff. Thus, no suspected ASMD cases were identified in the 244-neonate cohort.

## 4. Discussion

### 4.1. Developmental and Biological Determinants of ASM Activity

This study provides NICU-specific evidence that DBS-based ASM activity may be influenced by developmental maturity and early postnatal changes. ASM activity increased modestly with gestational age and birth weight and showed a substantial rise on repeat sampling in neonates with birth weight <2000 g. The lower ASM activity observed in preterm than term neonates (3.38 ± 0.90 vs. 4.16 ± 1.42 µmol/h/L; Welch’s *t*-test, *p* < 0.0001) further supports gestational maturity as a clinically relevant covariate. These observations complement emerging data from population newborn screening programs demonstrating that lysosomal enzyme activities vary with demographic and pre-analytic factors (e.g., age at collection, gestational age, and birth weight), motivating covariate-adjusted or age-stratified cutoffs and post-analytical tools to reduce false-positive results [16,29]. Since Japan’s routine NBS sampling occurs on postnatal days 4–6, NICU- and Japan-specific reference ranges are essential and should not be directly extrapolated from programs that collect DBS earlier (e.g., 24–48 h). However, because we did not include a healthy, non-NICU newborn reference cohort, the present data should be interpreted as NICU-specific rather than normative neonatal ASM values. Most published newborn-screening pilot studies have reported ASM screening performance using relative metrics such as percent daily mean activity (%DMA), daily median-based cutoffs, or multiple of the median (MoM), rather than absolute µmol/h/L values [16,17,18]. Although a Washington pilot study reported a mean ASM activity of 6.03 µmol/h/L in deidentified newborn DBS samples [22], direct comparison with our NICU cohort should be made with caution because of differences in study population, postnatal age at collection, and analytical platform. Prior DBS assay studies nonetheless suggest that leukocyte-related variables can influence lysosomal enzyme measurements, including a reported positive association between sphingomyelinase activity and lymphocyte number [26]. In addition, population-based newborn screening data indicate that demographic covariates such as gestational age, birth weight, and age at sample collection can affect DBS lysosomal enzyme activities more broadly [28]. ASMD guidelines also note that DBS ASM results may be influenced by anemia, leukopenia, and recent transfusions [3]. Together, these data support that the direction of effect observed in our NICU cohort is biologically plausible beyond the NICU setting, even if its magnitude may be accentuated in preterm or medically complex neonates.

Our hematological findings provide mechanistic and practical guidance for interpreting DBS ASM activity in medically complex neonates. The positive association with lymphocyte count and the negative association with hematocrit were consistent with ASM being enriched in nucleated blood cells but absent in erythrocytes. ASMD diagnostic and management guidelines and prior studies note that leukocyte composition, hematocrit, and transfusions can confound DBS enzyme assays [3,23,24,25,26,27]. By quantifying these relationships using confidence intervals, our study supports a hematology-informed interpretation and reinforces the value of repeat testing when low ASM results occur in the context of hematological instability.

### 4.2. Second-Tier Biomarkers for NICU ASMD Screening

Second-tier biomarkers such as lysosphingomyelin (LysoSM) and previously lysosphingomyelin-509 (PPCS) may improve the diagnostic specificity of ASMD screening [3,16,21,28]. Incorporating these biomarkers enhances specificity and enables the improved characterization of variants of uncertain significance, thereby facilitating a more accurate interpretation of molecular findings in screen-positive cases. For contextual interpretation, Polo et al. reported DBS LysoSM in unaffected controls with a median of 52.4 nmol/L and a 2.5th–97.5th percentile range of 6.6–112.8 nmol/L [28]. Against this background, the LysoSM concentrations observed in our three genetically confirmed ASMD patients (520–830 nmol/L) were clearly abnormal. Although budgetary constraints precluded second-tier biomarker measurements in the full cohort, analysis of three patients with genetically confirmed ASMD demonstrated markedly elevated LysoSM concentrations (520–830 nmol/L), consistent with the findings of Polo et al. [28]. Importantly, LysoSM elevation was independent of the degree of suppression of enzymatic activity (Table 1), suggesting that LysoSM quantification in DBS could serve as a robust second-tier test for reducing false-positive results in NICU populations. An approach that combines enzyme activity measurements with second-tier biomarkers may be particularly useful in NICU populations where biological variability is substantial. Future multicenter large-scale studies in NICU populations are required to systematically validate the clinical utility and optimal implementation of second-tier biomarkers within gestational age-adjusted screening frameworks.

### 4.3. Implications for ASMD Newborn Screening in NICU Populations

**Figure 6:**
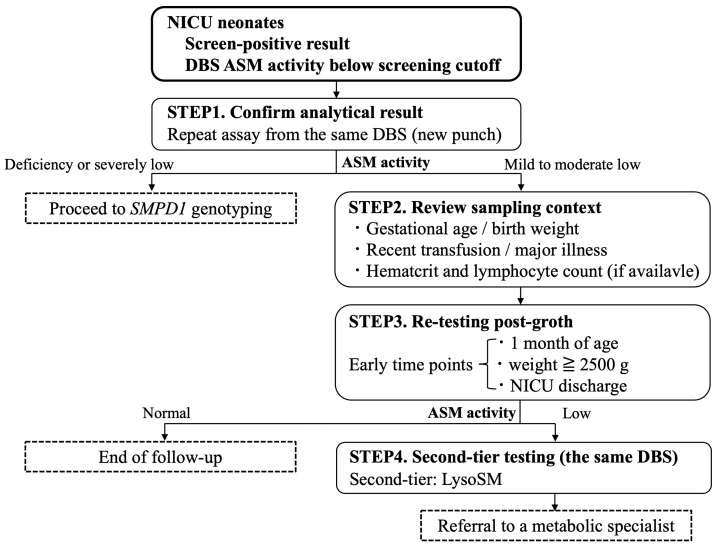
Proposed interpretation workflow for low DBS ASM activity in NICU newborn screening.

Given the dramatic therapeutic advances in olipudase alfa [8,9,10,11,12,13,14,15], early detection of ASMD through NBS has become clinically crucial. However, NICU populations have higher biological and pre-analytical variability, and our data showed that ASM activity is strongly influenced by the maturational status and hematological composition. Based on these findings and the existing international experience, we propose a practical, NICU-tailored interpretation workflow for low ASM activity in screen-positive NICU neonates (Figure 6).

- Confirmation of the analytical results and triage by severity: Repeat the ASM assay from the same DBS card (new punch) and review the internal quality control/sample quality. If the ASM is markedly low (deficiency range), proceed directly to *SMPD1* genotyping and urgent metabolic referral. If the ASM is mildly to moderately low, proceed to a clinical context review.
- Review of sampling context and likely confounders: Assess gestational age and birth weight, postnatal age at collection, and recent transfusion or major illness. When available, hematocrit and leukocyte/lymphocyte counts can contextualize low ASM activity and help distinguish physiologic suppression from true deficiency [3,23,24,25,26,27].
- NICU-tailored repeat DBS sampling after growth: For prematurity, birth weight < 2000 g, or hematological instability, obtain a repeat DBS at the earliest of approximately 1 month of age, birth weight ≥ 2500 g, or NICU discharge. If repeat ASM normalizes, document as physiologic/analytic low, and end the follow-up; if persistently low, proceed to second-tier testing and confirmatory evaluation.
- Second-tier biomarkers and confirmatory evaluation: Perform second-tier LysoSM on DBS to improve the specificity for ASMD [3,16,21,28]. If second-tier biomarkers are elevated and/or ASM remains low, refer the patient to a metabolic specialist to confirm the diagnosis based on leukocyte or skin fibroblast ASM activity, *SMPD1* genotyping, and integrated clinical assessment.

Flowchart summarizing NICU-tailored steps when the initial DBS ASM activity falls below the screening cut-off. After confirming the analytical result on a new punch from the same DBS card, markedly low values are triaged to immediate *SMPD1* genotyping; otherwise, sampling context is reviewed and repeat DBS is obtained after maturation (earliest of ~1 month of age, BW ≥ 2500 g, or NICU discharge). Persistently low results prompt second-tier LysoSM testing on DBS and a referral for confirmatory evaluation (leukocyte/skin fibroblast ASM activity, *SMPD1* genotyping, and clinical assessment). ASM, acid sphingomyelinase; ASMD, acid sphingomyelinase deficiency; DBS, dried blood spot; GA, gestational age; BW, birth weight; NBS, newborn screening; LysoSM, lysosphingomyelin.

### 4.4. Comparison with Previous Studies

Pilot programs in New York, Illinois, Italy, and other regions have established the feasibility of DBS-based ASM screening; however, direct quantitative comparison across studies remains difficult. Most programs reported screening performance using relative metrics such as %DMA, daily median-based cutoffs, or MoM rather than absolute ASM activity in µmol/h/L [16,17,18]. The Washington pilot study is one of the few reports to provide an absolute-value reference, describing a mean ASM activity of 6.03 µmol/h/L in deidentified newborn DBS samples [22]; however, population characteristics, postnatal age at sampling, and analytical platforms differed from those in our NICU cohort. Our study therefore extends the literature not by defining a normative newborn reference interval but by characterizing ASM activity in a physiologically complex NICU cohort and providing effect size estimates for developmental and hematological determinants. These results align with broader newborn screening experience showing that demographic variables and analytic drift can meaningfully influence enzyme-based screening and can be mitigated using age-stratified cutoffs, multiple of the median (MoM) approaches, and/or post-analytical tools [16,29]. The Illinois statewide experience further demonstrated high specificity, with screen-positive infants measuring 4–14% of the daily median and no false-positive screens identified [17]. Taken together, these data support the feasibility of enzyme-based ASMD screening in population programs while underscoring the added interpretive complexity of NICU cohorts, in whom prematurity, hematologic instability, and early postnatal maturation may shift enzyme activity distributions.

### 4.5. Strengths and Limitations

The strengths of this study include its relatively large NICU sample size (n = 244); standardized DBS processing and frozen storage; concurrent hematological data in a meaningful subset (n = 43); longitudinal measurements in neonates with birth weight <2000 g (n = 34); rigorous statistical methods with 95% CIs and effect sizes; and assay validation with demonstrated reproducibility (CV 3.9–9.7%).

However, this study has some limitations including the single-region, two-center design limiting generalizability of the results; limited representation of extremely premature neonates (<28 weeks, n = 1); absence of a healthy, non-NICU newborn reference cohort, which precluded direct establishment of normative neonatal DBS ASM values; absence of ASMD cases preventing sensitivity validation; hematological data being available for only 43 of 244 neonates because complete blood count testing at the NBS/DBS sampling timepoint was performed only when clinically indicated, and hematocrit analyses were restricted to same-day CBC-based measurements rather than pooled with blood gas-derived hematocrit values; and cross-sectional analyses limiting causal inference.

### 4.6. Implications for Clinical Practice and Future Research

ASM activity in NICU neonates could be shaped by developmental and hematological factors that may not be captured in community-based NBS cohorts. Incorporating these biological considerations into screening algorithms through NICU-specific reference ranges, hematology-informed interpretation, repeat testing protocols, and second-tier biomarkers will maximize the sensitivity and specificity of ASMD detection while minimizing false-positive results in this physiologically complex population.

Multicenter studies across Japan and internationally are needed to (1) confirm the generalizability of our findings; (2) establish calibrated NICU-specific cutoff values (potentially using MoM or covariate-adjusted post-analytical tools); (3) develop correction algorithms for hematological confounders; (4) prospectively validate two-step testing strategies; and (5) evaluate the utility of second-tier LysoSM-based biomarkers in reducing false positives and expediting confirmatory diagnosis in the NICU.

## 5. Conclusions

This first comprehensive characterization of DBS-based ASM activity in NICU-admitted neonates in Japan demonstrates that ASM activity is substantially influenced by developmental maturity (gestational age r = 0.219, *p* = 0.0006; birth weight r = 0.184, *p* = 0.0039) and hematological parameters (lymphocyte ρ = 0.394, *p* = 0.0089; hematocrit ρ = −0.372, *p* = 0.014). Longitudinal analysis demonstrated substantial postnatal increases (54.2% mean increase; 91.2% of neonates improved; Cohen’s d = 0.912), indicating that single fixed cutoffs are insufficient for interpreting ASM activity in medically complex newborns.

The effective implementation of ASMD newborn screening in NICU populations requires the following: (1) NICU-specific reference ranges accounting for developmental variability, (2) hematocrit and lymphocyte-adjusted interpretation algorithms, (3) two-step testing strategies with documented evidence that 91.2% of neonates with birth weights <2000 g show significant improvement on retest, and (4) second-tier LysoSM–based biomarkers for diagnostic specificity.

By defining ASM activity physiology in this unique population with quantified effect sizes and confidence intervals, this study provides foundational evidence for the development of robust ASMD screening algorithms that accommodate the distinct biological profiles of NICU neonates. These findings advance the establishment of expanded newborn screening for ASMD in Japan, ultimately enabling earlier diagnosis and intervention for affected individuals and optimizing outcomes through early enzyme replacement therapy.

## Figures and Tables

**Table 1:** *SMPD1* gene mutations and clinical phenotypes of the three patients with ASMD.

Case	Age/sex	Clinical Features	Genotype (cDNA)	Genotype (Protein)	ASM Activity (μmol/h/L)	LysoSM (nmol/L)	Phenotype
Patient 1	1 month F	Hepatic dysfunction	c.398G>A (homo)	p.C133Y (homo)	0.1	770	Type A
Patient 2	3 years F	Hepatomegaly, splenomegaly, Thrombocytopenia	c.[7del];[1144C>T]	p.[R3AfsTer74];[L382F]	0.4	830	Type B
Patient 3	54 years M	Hepatomegaly, splenomegaly, thrombocytopenia, respiratory failure	c.1480G>T (homo)	p.G494C (homo)	0.8	520	Type B

Notes: Variants were described according to the HGVS nomenclature using the *SMPD1* reference sequence NM_000543.5. ASM activity and LysoSM values were derived from dried blood spot samples. Patient 2 carried a null mutation (p.R3AfsTer74) on one allele but presented with a type B phenotype due to residual activity from the p.L382F allele. For contextual interpretation, published DBS LysoSM in unaffected controls was reported as a median of 52.4 nmol/L (2.5th–97.5th percentile, 6.6–112.8 nmol/L) in 253 controls [28]. Abbreviations: ASMD, acid sphingomyelinase deficiency; ASM, acid sphingomyelinase; cDNA, complementary DNA; fs, frameshift; LysoSM, lysosphingomyelin; Ter, termination codon; homo, homozygous.

**Table 2:** Clinical characteristics of the NICU cohort (n = 244), with hematological parameters available in a subset (n = 43).

		Range
**Gestational age (week)**	36.1 ± 2.1	(25–41)
**Birth weight (g)**	2353 ± 496	(773–4201)
**Sex (number) (%)**		
**Male**	134 (55%)	
**Female**	110 (45%)	
**White blood cells (/μL)**	9879 ± 3625	(4100–23,000)
**Lymphocyte (/μL)**	4601 ± 1739	(500–8050)
**Hematocrit (%)**	43.9 ±8.2	(31.8–63.0)
**Diagnosis (number)**		
**Low birth weight**	165	
**Respiratory failure**	138	
**Prematurity**	81	
**Hypoglycemia**	15	
**Heart disease**	8	
**Infection**	8	
**Jaundice**	5	
**Chromosomal abnormality**	2	

These diagnoses were not mutually exclusive. Blood data were obtained from 43 samples from which DBS and laboratory blood tests were performed on the same day.

## Data Availability

De-identified individual-level data for all 244 neonates (Supplementary Data S1), paired measurements for 34 neonates with birth weight <2000 g (Supplementary Data S2), and hematological parameters for 43 neonates with concurrent testing (Supplementary Data S3) are provided in the Supplementary Materials.

## Supplementary Materials

The following supporting information can be downloaded at: https://www.mdpi.com/article/10.3390/ijns12020022/s1. Supplementary Data S1: Individual-level clinical dataset for all 244 neonates; Supplementary Data S2: Longitudinal dataset with paired ASM activity measurements in 34 neonates with birth weight <2000 g; Supplementary Data S3: Hematological dataset for 43 neonates with concurrent blood sampling; Supplementary Tables S1 and S2: MS/MS operating conditions for ASM activity measurement.

## Abbreviations

The following abbreviations are used in this manuscript:

ASMD	acid sphingomyelinase deficiency
ASM	acid sphingomyelinase
NBS	newborn screening
NICU	neonatal intensive care unit
DBS	dried blood spot
CI	confidence interval
MoM	multiple of the median
