# Supplementary material for: Acid Sphingomyelinase Activity in Dried Blood Spot from Neonatal Intensive Care Unit–Admitted Neonates: A Pilot Study for Expanded Newborn Screening in Japan
Source: Int J Neonatal Screen. 2026 Apr 1;12(2):22. doi: 10.3390/ijns12020022 (PMC13108211; doi:10.3390/ijns12020022)
Supplement: Supplementary file 1 [file IJNS-12-00022-s001.zip › Supplementary Tables_kato.pdf]

Supplementary Table S1

Instrument parameters for the measurement of substrate, internal standard and product of ASM activity using MS/MS.

Interface	ESI (Electrospray ionization)
Polarity	Positive
Injection volume	2μL
Spray voltage	2.8 kV
Vaporizer temperature	0℃
Capillary temperature	270℃
Sheath gas pressure	25 L/h
Aux gas pressure	0 L/h
Analyzing mode	SRM (selected reaction monitoring)
Dwell time	0.1 s
Data format	Profile

Supplementary Table S2

Product ion, precursor ion, Stacked-Lens, collision energy and retention times for the fragmentation of substrate, product and internal standard using MS/MS.

Species	Precursor ion (*m/z*)	Product ion (*m/z*)	S-Lends RF amplitude voltage（V）	Collision energy（V）
ASM-S	563.4	184.0	130	20
ASM-P	398.4	264.3	90	17
ASM-IS	405.4	264.3	90	16

ASM, acid sphingomyelinase
